# Comparison of adhesion prevention capabilities of the modified starch powder-based medical devices 4DryField^®^ PH and Arista™ AH in the Optimized Peritoneal Adhesion Model

**DOI:** 10.7150/ijms.33277

**Published:** 2019-09-19

**Authors:** Daniel Poehnert, Lavinia Neubert, Juergen Klempnauer, Paul Borchert, Danny Jonigk, Markus Winny

**Affiliations:** 1 Department of General, Visceral and Transplantation Surgery, Hannover Medical School, Hannover, Germany; 2 Institute of Pathology, Hannover Medical School, Hannover, Germany

**Keywords:** Adhesion prevention, abdominal surgery, rat model OPAM, 4DryField^®^ PH, Arista^TM^ AH

## Abstract

Adhesion barriers can be based on numerous substances. In the rat Optimized Peritoneal Adhesion Model (OPAM) the starch-based hemostats 4DryField and Arista were tested for their capability to act in a preventive manner against adhesion formation (applied as a powder that was mixed *in situ* with saline solution to form a barrier gel). Adhesions were scored using the established scoring systems by Lauder and Hoffmann, as well as histopathologically using the score by Zühlke. Animals receiving saline solution were used as controls. As previously published, 4DryField reduced peritoneal adhesions significantly. However, Arista did not lead to a statistically significant reduction of adhesion formation. When comparing 4DryField and Arista applied in the same manner, only 4DryField was significantly effective in preventing peritoneal adhesions. Histopathological evaluations confirmed the results of the macroscopic investigation, leading to the conclusion that starch-based hemostats do not generally have the capability to function as effective adhesion prevention devices.

## Introduction

Surgery is the most common cause for formation of peritoneal adhesions. Predisposing factors include mechanical injury of the peritoneum and local ischemia due to manipulation and retraction of abdominal tissues during surgery 1-4. The incidence of postoperative adhesion formation ranges from 67 to 93% 5. Several adhesion prevention barrier agents addressing this problem are available on the market. In the majority of cases these agents function as a physical barrier to separate wound areas at risk of developing adhesions. These devices include adhesion barriers made from oxidized regenerative cellulose 6, polytetrafluoroethylene 7, icodextrin 8, hyaluronic acid/carboxymethyl cellulose 9 and starch 10. Typically, starch-based products are used solely as hemostats, such as Arista™ AH (Arista; Davol Inc., USA) 11. A unique starch-based medical device is 4DryField^®^ PH (4DryField; PlantTec Medical GmbH, Germany) as it is the only product proven to provide hemostasis and prevent the formation of adhesions. While 4DryField is applied as a powder for hemostasis, the powder is transformed into a gel by mixing with saline solution for adhesion prevention.

This raised the question if modified starch powders other than 4DryField might also be capable of reducing adhesion formation when applied in the same way as 4DryField. Previously, Hoffmann et al. 12 found Arista to be moderately effective in preventing adhesions, whereas no effect was observed in a study by Singh et al. 13. Therefore, the aim of the present study was to test 4DryField and Arista for their capability in preventing postoperative peritoneal adhesion formation in a challenging and well-reproducible rat model, the recently described Optimized Peritoneal Adhesion Model (OPAM) 14. This model has been shown to induce severest adhesions with high reliability and it has already been utilized successfully to examine the effectiveness of 4DryField compared to a control group 15, as well as in a comparative study with 4DryField and other adhesion prevention devices based on different materials 16. The model includes abrasion of the cecum and incision of the abdominal wall, as well as meso-stitch approximation of these lesions.

## Materials and Methods

### Animals

Thirty-six male Lewis rats were included in the study. They were housed under standard conditions, had access to fresh water at any time and were fed a standard diet ad libitum. Prior to and after surgery, daily monitoring of body weight and behavioral changes assessed animal welfare. Animal experiments were performed at the central animal laboratory of the Hanover Medical School, Germany, as well as the therapeutic experimental unit, Faculty of Medicine, Nantes, France. All protocols regarding animal life quality were conducted in accordance with national and European regulations. The present study was approved by The Lower Saxony State Office for Consumer Protection and Food Safety (LAVES Hannover, Germany; approval code 12/0751) and the Ethical Committee For Animal Experiments (CEEA) in Pays de la Loire, France (approved under the reference APAFIS9771).

### Surgical procedures and application of anti-adhesive agents

General anesthesia was achieved by ketamine (80 mg/kg body weight) and xylazine (5 mg/kg body weight) or inhalation of isoflurane 3%. The required level of narcosis was reached when the flexor reflexes were suppressed. A 3 cm long median laparotomy was performed after shaving and sanitizing the abdomen. Adhesion induction was carried out according to the OPAM 14: 1) the cecum was delivered and kept moist with a watery gauze swab, the cecal peritoneum was gently abraded repeatedly over a 1x2 cm area in a standard manner using a dry gauze until removal of visceral peritoneum resulted in sub-serosal bleeding and the creation of a homogenous surface of petechial hemorrhages; 2) the parietal peritoneum and inner muscle layer were sharply dissected in order to create a 1x2 cm abdominal wall defect; 3) both injured areas were approximated using a non-absorbable suture. Prior to surgery, animals were randomly assigned to one of the following three groups: control (n=10), 4DryField-treated (n=16) or Arista-treated (n=10, carried out in France). Control animals received 1.2 ml 0.9% sterile saline solution intraperitoneally. The two anti-adhesive agents 4DryField and Arista were each administered in a total amount of 300 mg powder/animal. The powder was evenly distributed on the two defects and then transformed into a gel by dripping with 1.2 ml sterile 0.9% saline solution before the approximating suture was placed. The abdomen was closed using a two-layer closure technique by consecutive sutures. Following surgery, the animals were monitored until they were completely awakened and kept warm using an infrared lamp. Animals received novaminsulfone or buprenorphine in a body-weight adapted dose to minimize postoperative pain. On postoperative day 7, the animals were sacrificed using CO_2_ narcosis followed by cervical disclosure. The peritoneal cavity was opened by an incision at a left-sided position remote to the original laparotomy scar to prevent damaging any potentially formed adhesions. Specimens of cecum, abdominal wall and adhesions were harvested for histopathological assessment.

A detailed protocol was generated and provided to the surgeons in France to ensure uniformity of execution and, thereby, comparability of the results. Apart from step-by-step descriptions of the procedures, photographs illustrated all steps in detail, particularly the abrasion of the cecum, the dissection of peritoneum and inner muscle layer, as well as the application of the adhesion barrier.

### Adhesion assessment

The adhesion formation between the defective abdominal wall and cecum was evaluated macroscopically by two independent observers according to the scoring systems by Lauder et al. 17 and Hoffmann et al. 12. The Lauder scoring system (Table 1) takes into account number, strength and distribution of adhesions in a single score, while the Hoffmann scoring system (Table 2) consists of three individual scores for area, extent and strength of adhesions that are summed up to yield a total score.

### Histology

Surgical specimens were fixed in buffered 4% formaldehyde solution. After dehydration and paraffin embedding, serial thin sections of 1-2 μm were mounted on glass slides, stained with standard Hematoxylin and Eosin (HE), Elastika-van-Gieson (EvG) and periodic acid-Schiff (PAS) staining (Sigma Aldrich Co Ltd, USA) and light microscope examinations were performed by experienced pathologists.

The quantitative analysis of the histologic stainings was performed using Zühlke's microscopic adhesion classification. This system has already been established for grading of peritoneal adhesions induced with models very similar to OPAM 19, 20.

### Statistical analyses

Adhesion scores are presented as arithmetic means with standard deviations (SD). Since most of the data sets did not follow a Gaussian distribution (as determined using the D'Agostino-Pearson normality test) the multiple comparisons of adhesion scores of the three groups were performed using Kruskal-Wallis test followed by Dunn's multiple comparisons test for non-parametric data (which utilizes correction for multiple comparison by statistical hypothesis testing). Groups were defined to be significantly different if p<0.05. Statistical analyses were performed using GraphPad Prism (Version 7.0b for Mac OS, GraphPad Software, Inc., La Jolly, USA).

## Results

All animals showed comparable viability and body weight development. None of the animals had to be sacrificed prematurely due to complications; all 36 animals completed the study.

### Adhesion development

**Figure 1:**
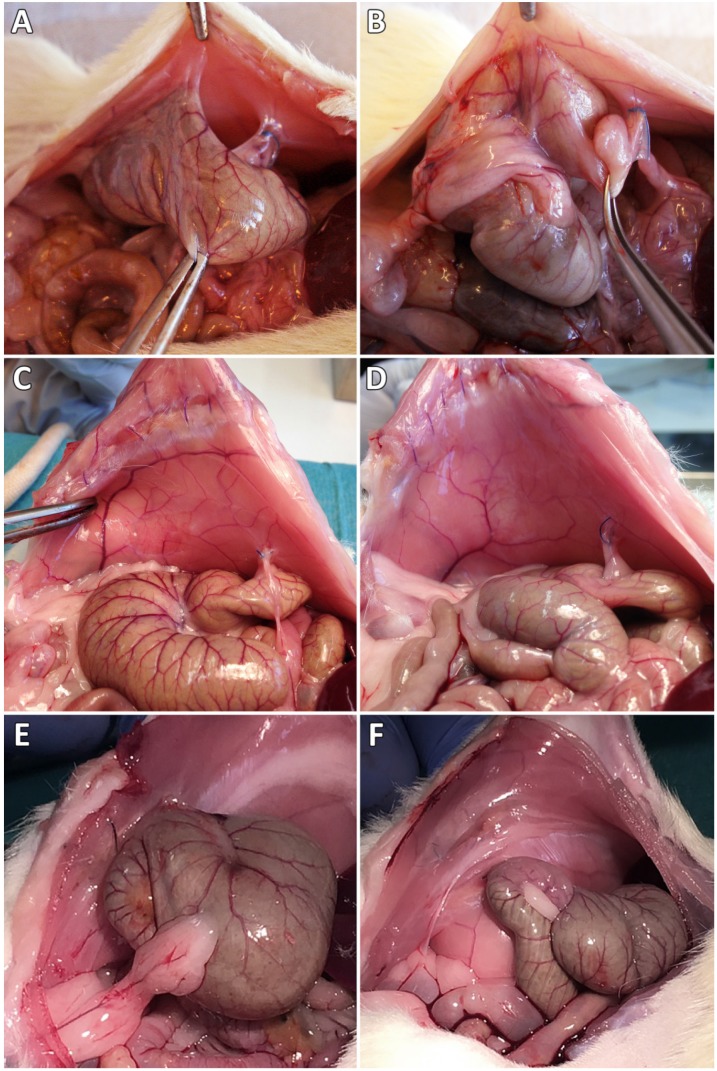
Representative photographs of the pathological evaluation of control (A,B), 4DryField- (C,D) and Arista-treated (E,F) rats on day 7.

In the control group, 9 of 10 animals showed peritoneal adhesions, which were rated with the maximum Lauder score, as well as the maximum scores regarding all of the Hoffmann categories (Figure 1A,B). None of the sixteen 4DryField-treated animals developed any adhesions (Figure 1C,D). In contrast, all 10 Arista-treated animals developed peritoneal adhesions (Figure 1E,F). Two developed filmy adhesions, with a Lauder score of 1 each. The total Hoffmann scores of these two animals differed and were 3 and 7, respectively. The other eight Arista-treated animals developed severe adhesions with Lauder scores of 4 (n=6) or 5 (n=2) and total Hoffmann scores of 8 (n=4), 9 (n=3) or 10 (n=1). The mean score value of each group was calculated and tested for significant differences (Table 3). Herein, 4DryField PH reduced the incidence and severity of peritoneal adhesion formation significantly compared to the control, as well as to the Arista-treatment group and concerning every evaluated scoring system. In contrast, Arista-treatment did not lead to a statistically significant reduction of adhesion formation in comparison to control animals.

### Histological Evaluation

**Figure 2:**
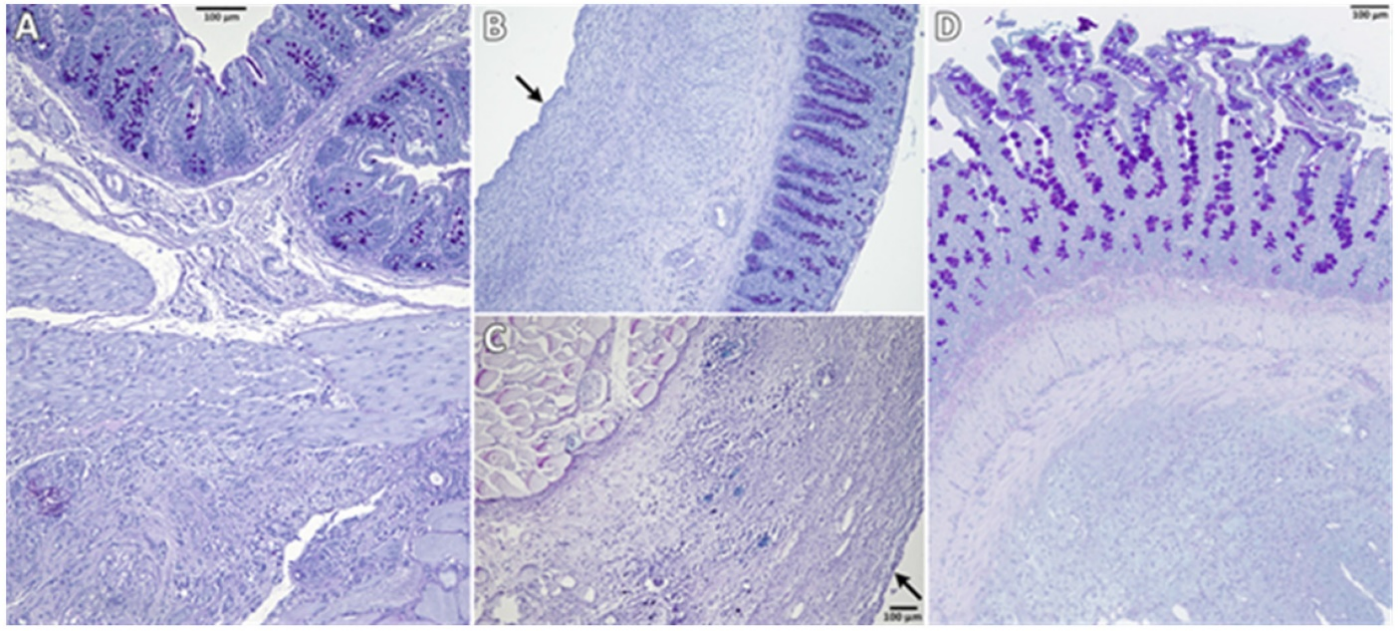
Representative histological slides (PAS-stained) of animals from the control (A), 4DryField (B, C) and Arista (D) groups. Black arrows indicate neomesothelial coverage.

Figure 2 shows representative PAS-stained tissue slides from all three groups. Figure 2A shows a control animal where the smooth muscle layers of the cecum (top) are fused to skeletal muscles of the abdominal wall (bottom) via dense granulating tissue. The histological findings support the macroscopic observation that both, cecum and abdominal wall, could not readily be separated by mechanical force. Figure 2B shows cecal and Figure 2C abdominal wall tissue of an animal from the 4DryField group.

In contrast to 9 of the 10 control animals, no agglutinations occurred in the 4DryField group. Furthermore, in all animals of the 4DryField group the lesions of the cecum and the abdominal wall defect had healed, and both featured neomesothelial cell coverage. The former abdominal wall defect was filled with fibrous tissue, which still contained slight remnants of 4DryField particles. Figure 2D shows an animal from the Arista group. As in Figure 2A the smooth muscles of the cecum (top) were fused to the skeletal muscles of the abdominal wall (bottom) via dense granulation tissue, preventing separation of cecum and abdominal wall by mechanical force.

The microscopic classification of the adhesions according to Zühlke et al. 18 was performed all animals. In the control group one animal was scored 0, two were scored 3 and seven were scored 4, in the 4DryField group the microscopic assessment was equivalent to the macroscopic investigation with all 16 animals being scored 0. In the Arista group one animal was scored 1, one was scored 2, four were scored 3, and four were scored 4.

Like for the macroscopic adhesion assessment the mean scores were calculated and tested for significant differences (Table 5). The results were conform with the macroscopic assessment, the 4DryField treated animals scored significantly better results than the Arista treated ones as well as the control animals, while the Arista group did not show statistically significant differences to the control.

## Discussion

As shown in previous studies 14-16, the OPAM consistently induced severe peritoneal adhesions after cecal abrasion and creation of abdominal wall defects in rats.

4DryField revealed excellent adhesion prevention capabilities, completely preventing the formation of any adhesions. Furthermore, a newly-formed mesothelial layer was found by histopathological assessments of the previously injured sides. 4DryField could be shown to be highly effective in preventing peritoneal adhesions in previous studies, being prophylactically applied either as a preformed gel or as powder that was transformed *in situ* into a gel by adding saline solution 15, 16.

The adhesion prevention capabilities of Arista were examined for the first time in 2009 by Hoffmann et al. 12. Although the authors found the adhesion development to be significantly reduced in comparison to a control group, the adhesion reduction was still limited with an adhesion score of 3.9 (Arista) *vs*. 6.0 (control). In 2013, Singh et al. challenged these results in a randomized-controlled trial using Arista in a rat model with adhesion induction at the cecum and the uterine horn. Adhesion prevention capabilities of Arista were found to be not different from those of the control group, which received Ringer's lactate solution 13.

In our present study, Arista did not lead to a statistically significant reduction of adhesion formation compared to control animals using Lauder and Hoffmann scoring systems, as well as systematic histopathological examinations using the Zühlke microscopic classification system and confirming the macroscopic results. The microscopic analysis showed tight agglutinations of cecum and abdominal wall via granulating tissue, comparable to those of the control animals. When comparing 4DryField and Arista applied in the same manner, 4DryField resulted in a significantly more effective reduction of adhesion scores.

Limited comparability of the results arising from differing surgical performance at the two study centers can be excluded due to strict monitoring of the comparability as described above. Additionally, the OPAM has been used at the Hanover Medical School extensively 14-16 and different surgeons have performed surgeries following this protocol in the past, but a correlation of results with the respective surgeon has never been observed. Correspondingly, the model has been shown to be highly reliable and very robust.

In summary, in this experimental animal model of severe peritoneal adhesion induction only 4DryField but not Arista was effective in reducing postoperative adhesion formation when both devices were applied in the same manner. Our results show that modified starch-based powder hemostats are not naturally capable to reduce the formation of peritoneal adhesions. Instead, the effectiveness depends on the specific properties of the individual product, which are often not reported in detail and might be of interest for further investigations.

## Figures and Tables

**Table 1:** Adhesion scoring system according to Lauder et al. 17

Score	Description
0	No adhesions
1	Thin filmy adhesions
2	More than one thin adhesion
3	Thick adhesion with focal point
4	Thick adhesion with planar attachment
5	Very thick vascularized adhesions or more than one planar adhesion

**Table 2:** Adhesion scoring system according to Hoffmann et al. 12

Score	Description
**Area score**	
0	No adhesion
1	Cecum to bowel adhesion
2	Cecum to sidewall adhesion over less than 25% of the abraded surface area
3	Cecum to sidewall adhesion between 25 and 50% of the abraded surface area
4	Cecum to sidewall adhesion over more than 50% of the surface area
**Strength score**	
0	No adhesion
1	Gentle traction required to break adhesion
2	Blunt dissection required to break adhesion
3	Sharp dissection required to break adhesion
**Extent score**	
0	No adhesion
1	Filmy adhesion
2	Vascularized adhesion
3	Opaque or cohesive adhesion

**Table 3:** Microscopic adhesion classification according to Zühlke et al. 18

Score	Description
0	No adhesions
1	Weak connective tissue, rich cell, new and old fibrin, thin reticulin fibrils
2	Connective tissue which has cells and capillaries. few collagen fibers
3	Thicker connective tissue. Few cells and elastic and smooth muscle fibers, more vessels
4	Old and thick granulation tissue, poor cells, difficult separation of serosal surfaces

**Table 4:** Arithmetic mean values (AM), standard deviations (SD) and p-values in comparison to the control (p (ctrl)) or 4DryField (p (4DF)) groups (statistically significant difference if p<0.05, *)

Score	Group	AM	SD	p (ctrl)	p (4DF)
	control	4.5	1.6		
Lauder	4DryField	0.0	0.0	<0.0001*	
	Arista	3.6	1.4	0.6512	0.0008*
	control	3.6	1.3		
Hoffmann Area	4DryField	0.0	0.0	<0.0001*	
	Arista	2.4	0.8	0.4556	0.0013*
	control	2.7	0.9		
Hoffmann Strength	4DryField	0.0	0.0	<0.0001*	
	Arista	2.7	0.7	>0.9999	<0.0001*
	control	2.7	0.9		
Hoffmann Extent	4DryField	0.0	0.0	<0.0001*	
	Arista	2.8	0.6	>0.9999	<0.0001*
	control	9.0	3.2		
Hoffmann Total	4DryField	0.0	0.0	<0.0001*	
	Arista	7.9	1.9	0.4565	0.0013*

**Table 5:** Arithmetic mean values (AM), standard deviations (SD) and p-values in comparison to the control (p (ctrl)) or 4DryField (p (4DF)) groups (statistically significant difference with p<0.05, *)

Score	Group	AM	SD	p (ctrl)	p (4DF)
	control	3.4	1.3		
Zühlke	4DryField	0.0	0.0	<0.0001*	
	Arista	3.1	1.0	>0.9999	0.0001*
